# Mayer-Rokitansky-Kuster-Hauser Syndrome Associated with Severe Inferior Vena Cava Stenosis

**DOI:** 10.1155/2014/745658

**Published:** 2014-07-23

**Authors:** Laura Londra, Kyle Tobler, John Wu, Lisa Kolp

**Affiliations:** Division of Reproductive Endocrinology and Infertility, Department of Gynecology and Obstetrics, Johns Hopkins University School of Medicine, 10751 Falls Road, Suite 280, Lutherville, MD 21093, USA

## Abstract

*Precis*. The postoperative course of a neovagina creation procedure in a young woman with Meyer-Rokitansky-Kuster-Hauser syndrome was complicated, despite prophylaxis, by extensive pelvic deep venous thrombosis secondary to unsuspected severe inferior vena cava stenosis. *Background*. Mayer-Rokitansky-Kuster-Hauser (MRKH) syndrome is characterized by congenital vaginal agenesis and an absent or rudimentary uterus in genotypical females. Malformations of the inferior vena cava (IVC) are not commonly associated with MRKH syndrome. We report a case of a patient with MRKH syndrome with severe IVC stenosis that was diagnosed when the patient presented with extensive pelvic deep venous thrombosis (DVT) during the postoperative course of a neovagina creation. *Case*. A 19-year-old female underwent a McIndoe procedure. Despite DVT prophylaxis, extensive pelvic DVT of the femoral vein was diagnosed on postoperative day 7. Therapeutic anticoagulation was initiated, and pharmacological and mechanical thrombolysis were performed. During these procedures, a hypoplastic IVC was noted. *Conclusion*. MRKH syndrome can be associated with IVC malformations, which constitute an anatomical risk factor for postoperative DVT.

## 1. Introduction

Mayer-Rokitansky-Kuster-Hauser (MRKH) syndrome is a rare phenomenon characterized by congenital absence of the vagina with variable uterine development. The incidence is approximately 1/4500. The most common patient presentations are primary amenorrhea and inability to have intercourse [1]. MRKH syndrome is classified as either type I, with isolated Müllerian anomalies, or type II, with associated malformations in other systems. Associated malformations can involve kidneys, skeleton, hearing, extremities, facies, and cardiovascular systems [1, 2]. We report the case of a 19-year-old woman who initially presented with type I MRKH syndrome. During the postoperative course of a neovagina procedure, the patient developed extensive pelvic deep venous thrombosis (DVT) and was ultimately diagnosed with a previously unsuspected inferior vena cava (IVC) malformation. Anomalies of the infrarenal IVC have not been previously reported in association with MRKH syndrome.

## 2. Case

A 19-year-old female was referred to our clinic for evaluation of primary amenorrhea and suspicion of MRKH syndrome. Pelvic examination revealed the absence of a vagina, and abdominal ultrasound showed a small midline pelvic structure consistent with a rudimentary uterus with no endometrial stripe and ovaries that appeared normal. A review of the patient's medical records revealed a pelvic magnetic resonance imaging (MRI) report that described findings consistent with bilateral, normal appearing kidneys and no anatomical abnormalities. It is noteworthy that the MRI images were not available and there was no description of the appearance of the IVC in the report. The patient had a history of renal superior mesenteric vein and splenic vein thromboses in early infancy, with abdominal vascular calcifications. These calcifications were noted on abdominal X-ray and ultrasound during a work-up for gastrointestinal symptoms of difficulty with feeding and vomiting at 20 days of age. A review of medical records from this early infancy time period showed a pediatric cardiology consultation that confirmed a normal heart on echocardiogram. In addition, a thrombophilia work-up, including activated protein C resistance, antithrombin level, protein C and S levels, antiphospholipid antibodies, homocysteine serum concentration, and factor-V Leiden mutation, was negative. The patient had no further symptoms after the first month of life. She had no history of tobacco use or hormonal therapy.

The initial management for this patient was with vaginal dilators, which she attempted for one year. The patient was ultimately unable to create a functional vagina and refused to continue with the use of dilators. Surgical options were discussed, and the patient elected to undergo a modified Abbe-McIndoe technique. The procedure included the harvesting of a skin graft from the upper thigh, the creation of the vaginal recipient site, crafting of the neovagina over a silicone vaginal stent (Silimed vaginal stent, Silimed, Inc., Dallas, TX), and subsequent placement of the stent [3]. The procedure was uncomplicated, and postoperative care included strict bed rest, DVT prophylaxis with low molecular weight heparin (LMWH) at 40 mg sq daily, and sequential compression devices on the lower extremities. On postoperative day seven, a routine exam for replacement of the stent was performed. The neovagina tissue graft was noted to be healing appropriately. However, swelling and erythema were subsequently noted on the upper right thigh, along with patient's complaint of right side extremity pain. An evaluation of the lower extremities by venous Doppler revealed no evidence of flow or compressibility within the right common femoral and femoral veins, with an occlusive thrombus extending into the greater saphenous vein.

A therapeutic protocol for anticoagulation with unfractioned heparin drip was instituted immediately, resulting in an excellent response of hematological parameters. Due to the extensive thrombosis, and in consultation with the Hematology and Interventional Radiology Departments, pharmacological and mechanical thrombolysis on the right common iliac, external iliac, common femoral, and superficial femoral veins were performed with tissue plasminogen activator and a Bacchus Trellis (Bacchus Vascular, Inc., Santa Clara, CA) device, respectively. Suction thrombectomy was performed without complications in the iliac and femoral veins, as described elsewhere [4].

Venograms of the right common iliac, external iliac, common femoral, superficial femoral, and popliteal veins were performed after completion of the thrombectomy. No patent IVC was identified. Extensive collateral flow through lumbar veins, consistent with a congenital absence or chronic occlusion of the IVC, was described. A computed tomography (CT) image of the abdomen and pelvis was acquired after intravenous administration of nonionic contrast on the day after the thrombolysis with the goal of clarifying the anatomical findings regarding the IVC that were found during the thrombectomy procedure. The CT image identified an infrahepatic IVC with diminutive caliber along with prominent azygos and lumbar veins.

The patient had significant improvement within 24 hours and was discharged within 3 days following the thrombectomy procedure. The anticoagulation plan was continued for 6 months and the patient did well.

## 3. Comment

This patient's presentation was notable because of the extensive nature of proximal pelvic DVT that occurred despite aggressive prophylaxis and because of the diagnosis of severe hypoplasia of the IVC in association with MRKH syndrome. The fact that the modified Abbe-McIndoe procedure was a pelvic surgery with a requirement for strict postoperative bed rest for 7 days is noteworthy because the bed rest would clearly have added to the DVT complication. In addition, the patient's history of mesenteric vein calcification in infancy may also be related to the present diagnosis of possible chronic occlusion of the IVC and/or an IVC with a diminutive caliber coupled with prominent azygos and lumbar veins. Although MRI of the pelvis has long been established as a useful imaging technique in the diagnosis of anomalies associated with MRKH syndrome [5], its utility in diagnosing cardiovascular-associated anomalies was less clear in this case. An MRI was not performed because of the lack of suspicion of a major vascular anomaly that would have prompted a more extensive evaluation including the upper abdomen.

The MRKH syndrome develops* in utero *between the 4th and 12th week of pregnancy. Multiple organ systems have been reported to be associated with the MRKH syndrome spectrum, including kidneys, skeleton, auditory system, extremities, facies, and cardiovascular system [6]. The coexistence of IVC anomalies with previously described associated urological anomalies is therefore possible. As an example, horseshoe kidneys are associated with a higher frequency of renal vein and vena cava anomalies [7, 8].

Nevertheless, this patient had normal appearing kidneys. Furthermore, to our knowledge, IVC hypoplasia has not been reported among the cardiovascular malformations associated with MRKH. A PubMed search using the words “Meyer-Rokitansky-Kuster-Hauser” and “cardiovascular anomalies” returned three reports, including one case of partial anomalous pulmonary drainage [9], one case of total anomalous pulmonary venous return [10], and one case (only abstract available in English) consistent of aortic isthmus stenosis [11]. It is plausible that the IVC anomaly described in our case report was unrelated to the Müllerian anomaly underlying MRKH syndrome and rather was a coincidental diagnosis that went overlooked in the MRI report that we obtained with the patient's medical records. We only had access to the MRI report and not the preoperative MRI images, so we are unable to determine if an IVC anomaly was overlooked.

Vena cava development is a complex process involving the formation and regression of parts of three complete venous systems. Interruption or congenital stenosis of the IVC consists of a developmental defect of the IVC and collateral circulation that occurs most commonly via the azygos-hemiazygos system. The presence of symptoms is highly dependent on the presence of a well-developed azygos-hemiazygos continuation. In a review of 7,972 CT scans of patients 14 years or older, the prevalence of interruption or congenital stenosis of the IVC in patients who had undergone multidetector row computed tomography (MDCT) was estimated to occur in 0.15% of cases [12]. The most common cause of presenting symptoms was DVT in patients of a relatively young age (range, 14–30 years). Well-developed azygos-hemiazygos continuation was observed in all patients who were asymptomatic, as was the case of our patient. The presence of acute or recurrent DVT, diffuse venous collaterals, including abdominal wall, varices, varicocele, and hemorrhoids, or venous aneurysm in a relatively young patient may be an indication to use CT or MRI for the diagnosis of interruption or congenital stenosis of the IVC.

The postoperative outcome of our patient after the DVT diagnosis was notable with regard to the excellent response to anticoagulation. However, the persistence of symptoms and extension of the thrombosis prompted a discussion with the patient about risks and benefits of thrombolysis and thrombectomy procedures. The procedure is usually highly effective [13], but the risk of bleeding in the setting of recent surgery needs to be carefully weighed against the benefits of mitigation of thrombotic complications. In this case, MRKH syndrome was unusually associated with severe, unsuspected IVC congenital hypoplasia which presented a risk factor for postoperative DVT, despite thromboprophylaxis, as well as for pelvic surgery complications because of the associated anatomy distortion and extensive collateral venous development. Thrombolysis with thrombectomy procedures should be carefully considered for shortening the course of DVT resolution in selected patients.
